# Supplementary figures and images for: Co-targeting menin and LSD1 dismantles oncogenic programs and restores differentiation in MLL-rearranged AML
Source: bioRxiv. 2025 Oct 15:2025.10.13.681683. Preprint. [Version 1] doi: 10.1101/2025.10.13.681683 (PMC12632788; doi:10.1101/2025.10.13.681683)

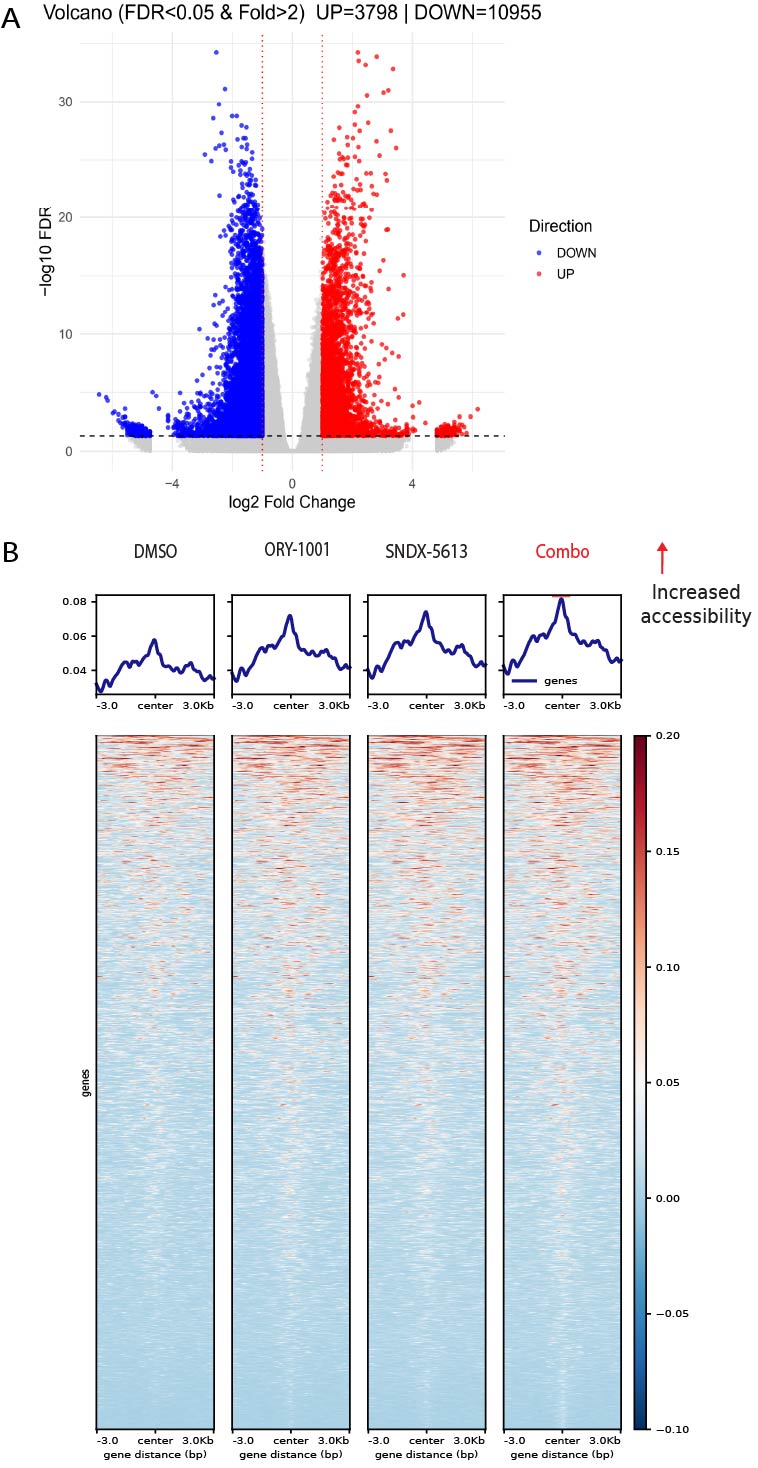

Supplement: Supplement 1 [file media-1.jpg]

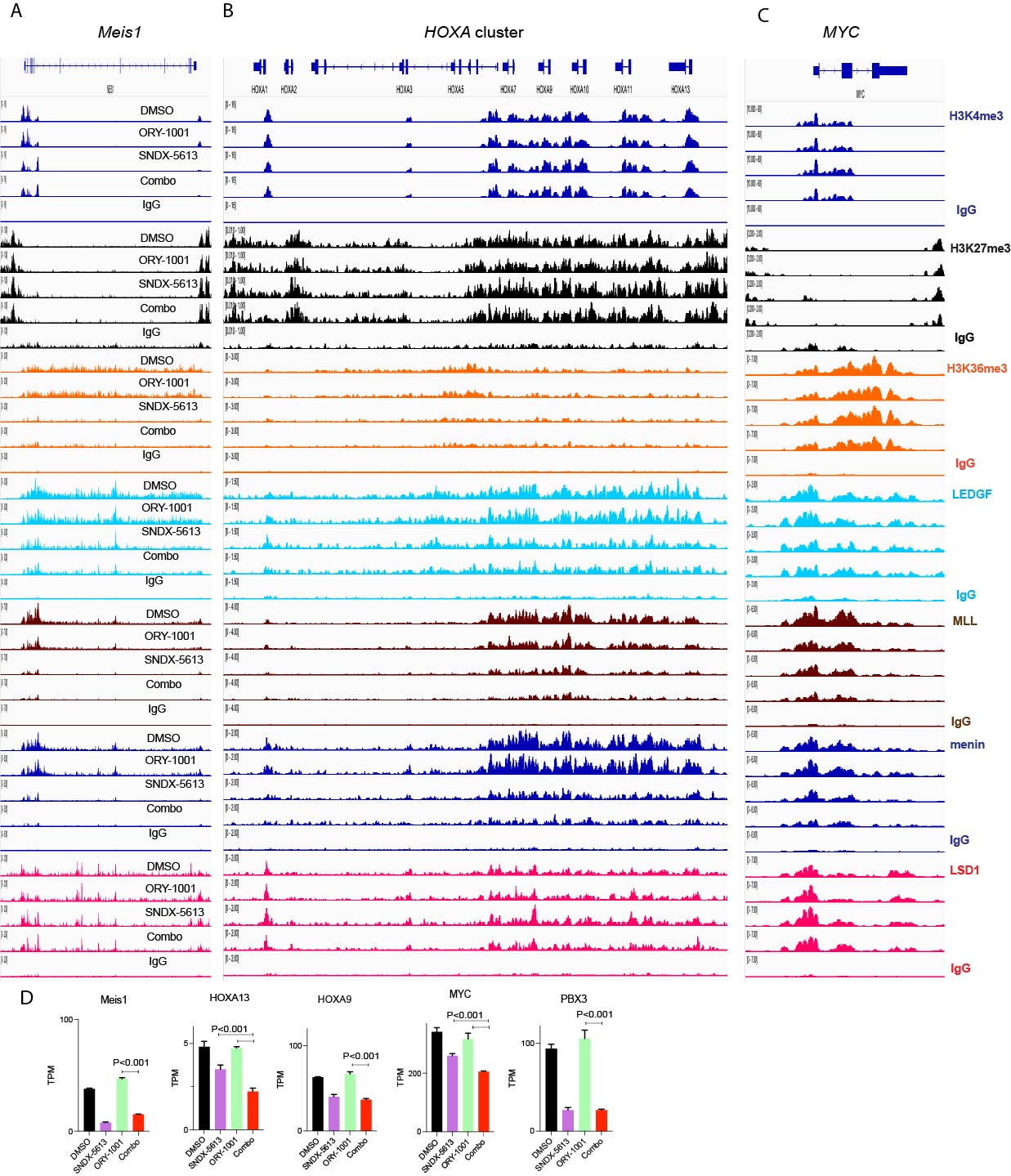

Supplement: Supplement 2 [file media-2.jpg]

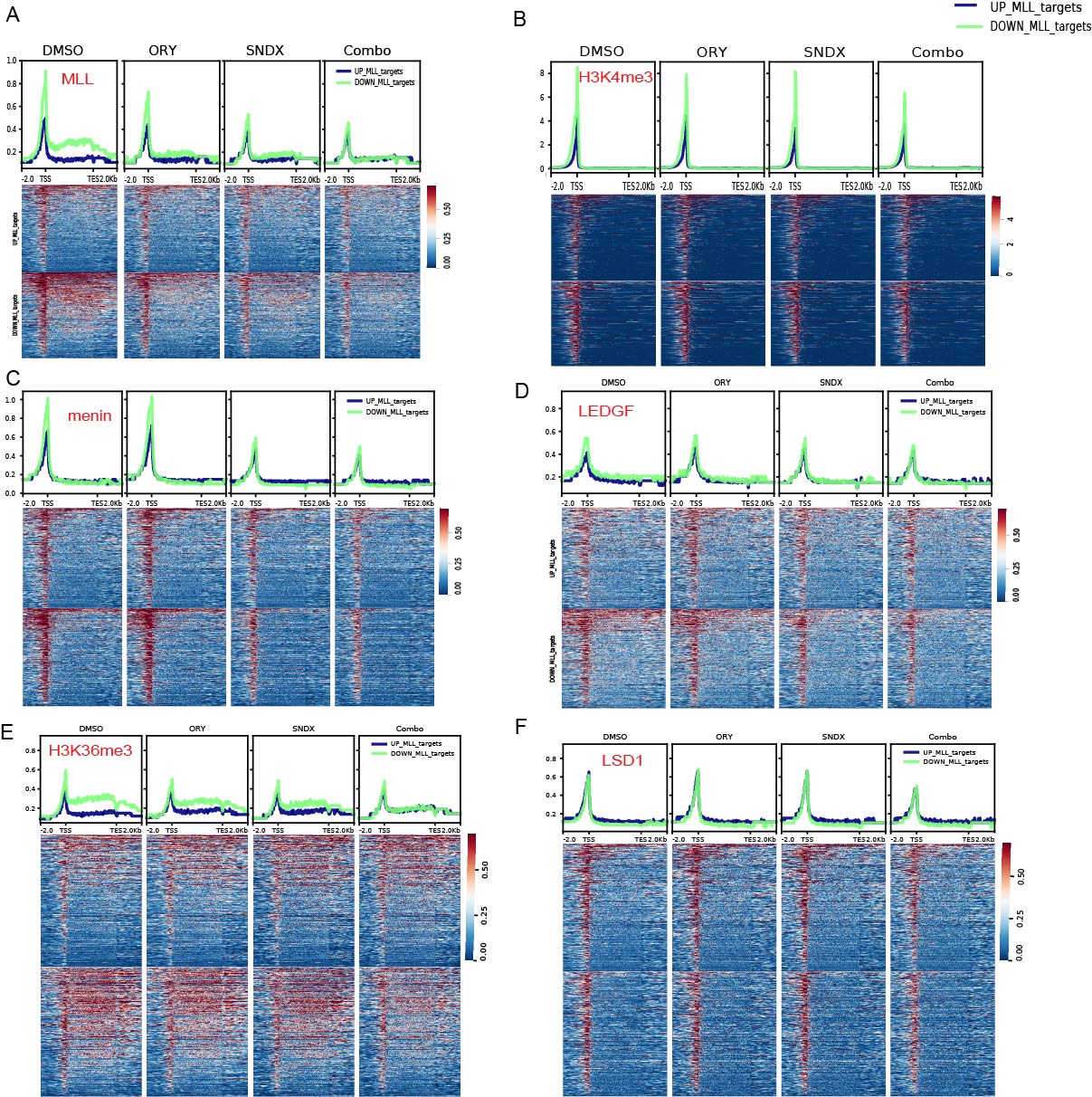

Supplement: Supplement 3 [file media-3.jpg]
